# Cryoballoon ablation for atrial fibrillation in patients with heart failure and reduced left ventricular ejection fraction: A systematic review and meta‐analysis

**DOI:** 10.1002/clc.24177

**Published:** 2023-10-25

**Authors:** Amira M. Taha, Nada I. Hendi, Ahmed B. Elwekel, Ahmed Atia, Nouran A. Taha, Abhigan B. Shrestha, Mohamed Elbanna

**Affiliations:** ^1^ Faculty of Medicine Fayoum University Fayoum Egypt; ^2^ Faculty of Medicine Ain Shams University Egypt; ^3^ Faculty of Medicine Al‐Azhar University Cairo Egypt; ^4^ Faculty of Medicine Cairo University Cairo Egypt; ^5^ MARS‐Global London UK; ^6^ M Abdur Rahim Medical College Dinajpur Bangladesh

**Keywords:** atrial fibrillation, cryoablation, cryoballoon, cryotherapy, heart failure

## Abstract

The coexistence of atrial fibrillation (AF) with heart failure (HF) is prevalent, leading to severe complications. This review aimed to investigate the success rate and efficacy of cryoballoon ablation (CBA) by measuring the improvement in the New York Heart Association (NYHA) classification and the effect on the left ventricular systolic function in patients with AF accompanied by heart failure with reduced ejection fraction (HFrEF). Electronic databases search included PubMed, Web of Science, and Scopus in January 2023. Outcomes addressed the following: left ventricular ejection fraction (LVEF) improvement, AF recurrence, NYHA classification improvement, and mortality. STATA 17.0 software was used for data analysis. The effect size for the studies was a standard mean difference (SMD) with a 95% confidence interval (CI) for outcomes. Proportion analysis with 95% CI was used for freedom from early AF and AF after 2 years and all‐cause death. We included six studies, including 1699 HF patients with 365 HFrEF patients. The SMD of postoperative LVEF compared to preoperative LVEF in HFrEF was 0.99 ([95% CI: 0.60, 1.39], *p* = .00), and for NYHA was −1.12 ([95% CI: −1.36, −0.87], *p* = .00). The analysis results in HFrEF patients for freedom from AF after 1 year was 65% ([95% CI: 0.55, 0.75], and after 2 years was 39% ([95% CI: 0.10, 0.67]). Proportional analysis was conducted for all‐cause death, resulting in 3% mortality ([95% CI: −0.01, 0.07]). Cryoablation of AF accompanied by HFrEF appeared safe as it reduced AF recurrence and enhanced clinical outcomes.

## Graphical abstract

Overview of cryoballoon ablation for atrial fibrillation in patients with heart failure and reduced left ventricular ejection fraction

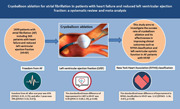

**Abbreviations**
AF: atrial fibrillation
CAD: carotid artery disease
CBA: cryoballoon ablation
CI: confidence interval
DM: diabetes mellitus
HF: heart failure
HFmrEF: heart failure with mildly reduced ejection fraction
HFpEF: heart failure with preserved ejection fraction
HFrEF: heart failure with reduced ejection fraction
HTN: hypertension
LVEF: left ventricular ejection fraction
NYHA: New York Heart Association

## 1 INTRODUCTION

In the next 50 years, the central focus of cardiovascular (CV) care is anticipated to converge mainly on two disorders: heart failure (HF) and atrial fibrillation (AF). The prevalence of these conditions is increasing, resulting in high mortality rates and medical costs. Their close relationship is based on common underlying causes and risk factors. They are closely related due to shared underlying causes and risk factors.^1^ International guidelines classify HF into three subcategories: heart failure with reduced left ventricular ejection fraction (HFrEF; ejection fraction [EF] < 40%), heart failure with preserved left ventricular ejection fraction (HFpEF; EF > 50%), and HF with mildly reduced left ventricular ejection fraction (HFmrEF; EF 40%–49%).^2^ AF not only contributes to the development of HF but also worsens systolic and diastolic functions.^3^ It is associated with a threefold increase in the incidence of new‐onset HF. Conversely, structural and hormonal alterations in the hearts of individuals with HFrEF and HFpEF markedly elevate the propensity for the development and progression of AF. The prognosis for HF and AF patients is decidedly unfavorable, irrespective of which condition manifested initially.^4^

The paucity of viable therapeutic options for AF in HF patients is notable, as most antiarrhythmic medications are either contraindicated or poorly tolerated. Hence, catheter ablation has emerged as a favored treatment for rhythm control. Predominantly, there are two principal forms of catheter ablation: radiofrequency ablation (RFA) and cryoballoon ablation (CBA).^5^ Numerous studies have shown that RFA reduces mortality and hospitalization in AF patients with HFrEF.^6^ In a multicenter trial called CASTLE‐AF, HF patients were randomly assigned to receive either medical treatment or RF ablation. The study found that RF ablation significantly decreased all‐cause mortality and HF hospitalizations compared to medical therapy. However, RF ablation is technically complex, requiring a steep learning curve and lengthy treatment time.^6^

On the other hand, CBA technology has emerged as a more straightforward approach to pulmonary vein isolation. While data on CBA in HF patients is somewhat limited,^7^ the FIRE and ICE trial showed potential benefits such as reduced CV rehospitalizations, fewer electrical cardioversions and repeat ablations compared to RF catheter ablation, faster technique, and fewer complications. The FIRE and ICE trial also demonstrated some potential advantages of CBA for HF patients, such as a decrease in CV rehospitalizations, fewer electrical cardioversions and repeat ablations compared to RF catheter ablation, absence of additional fluid burden from the irrigation catheter, a faster technique, and fewer complications.^8^ Moreover, catheter ablation significantly lowered all‐cause mortality in the subset of patients having both AF and HF, according to an analysis of the recent CABANA trial.^9^

Nonetheless, there remains an information deficit concerning the employment of second‐generation cryoballoon‐based pulmonary vein isolation in HFrEF patients. This review aims to investigate the success rate, safety, and effectiveness of CBA in improving clinical outcomes such as the New York Heart Association (NYHA) classification improvement and its impact on left ventricular systolic function in AF patients with HFrEF.

## 2 METHODS

This systematic review and meta‐analysis were implemented using the Preferred Reporting Items for Systematic Reviews and Meta‐Analyses (PRISMA) statement guidelines, and all steps were done with tight compliance with the Cochrane Handbook of Systematic Reviews and Meta‐analysis.^10^, ^11^

### 2.1 Literature search strategy

The following medical electronic databases were checked: PubMed, Web of Science, and Scopus, and a manual screening of all relevant articles through January 2023. Our search strategy included the following terms (“Atrial Fibrillation” OR “Atrial Fibrillations” OR “AF”) OR (“Cryosurgery” OR “Cryotherapy” OR “Cryoballoon” OR “Cryothermal”) OR (“Heart failure” OR “Cardiac failure” OR “Heart decompensation” OR “Myocardial failure”). There were no limitations, time restrictions, or any other filters applied. The detailed search strategy is present in File S1.

### 2.2 Study selection

We performed the screening process using Rayyan software.^12^ Two independent authors did the screening in two steps: Title/Abstract screening and full‐text screening. Any conflict was resolved by consensus or by referring to a third author. We included both randomized clinical trials and observational studies with the following criteria: a demographic of patients with AF together with HFrEF, an intervention which is cryoablation, and finally, the outcome which is at least one of the following: left ventricular ejection fraction (LVEF) improvement, freedom from AF recurrence, NYHA classification improvement, mortality, and complications. Studies were excluded if they were not published in English. Additionally, we excluded protocols and conference abstracts.

### 2.3 Methodological quality assessment

We assessed the included studies' potential risk of bias using the ROBINS‐1 tool, which includes the following domains: selection bias, confounding bias, intervention classification bias, incomplete outcome data, outcome measurement, and reporting bias.^13^ Our judgment was categorized into low, moderate, serious, or critical risk of bias.

### 2.4 Data extraction

Using a Google Sheet, two authors retrieved data from the included publications. The data extraction sheet included the following: study design, country, intervention, follow‐up period, patient characteristics, and main outcomes. Additionally, we extracted procedural data, including procedure time, number of isolated pulmonary veins, repeated ablation, and complications.

### 2.5 Strategy for data synthesis and statistical analysis

The data of included studies were pooled and analyzed with STATA 17.0 (StataCorp) software. The effect size for the studies was standard mean difference (SMD) with a 95% confidence interval (CI) for outcomes post‐ and preoperative difference for LVEF and NYHA, respectively. Additionally, proportion analysis with 95% CI was used for freedom from early AF and AF after 2 years and all‐cause death. Heterogeneity was considered significant when Cochrane *Q* test I2 statistic >50% and statistically significant when two‐tailed *p* < .05. The “dersimonian laird” random effect model was adapted to pool the calculations.

The association of other parameters like comorbidities (hypertension [HTN], diabetes mellitus [DM], carotid artery disease [CAD]), age, sex, follow‐up period, and persistent AF was considered in the regression analysis. Sensitivity analysis was examined to determine each study's impact on the pooled analysis. Finally, publication bias was plotted with a funnel plot using the regression‐based Eggers test.^14^ Publication bias was deemed significant at a *p* < .1.

## 3 RESULTS

### 3.1 Study selection

**Figure 1:**
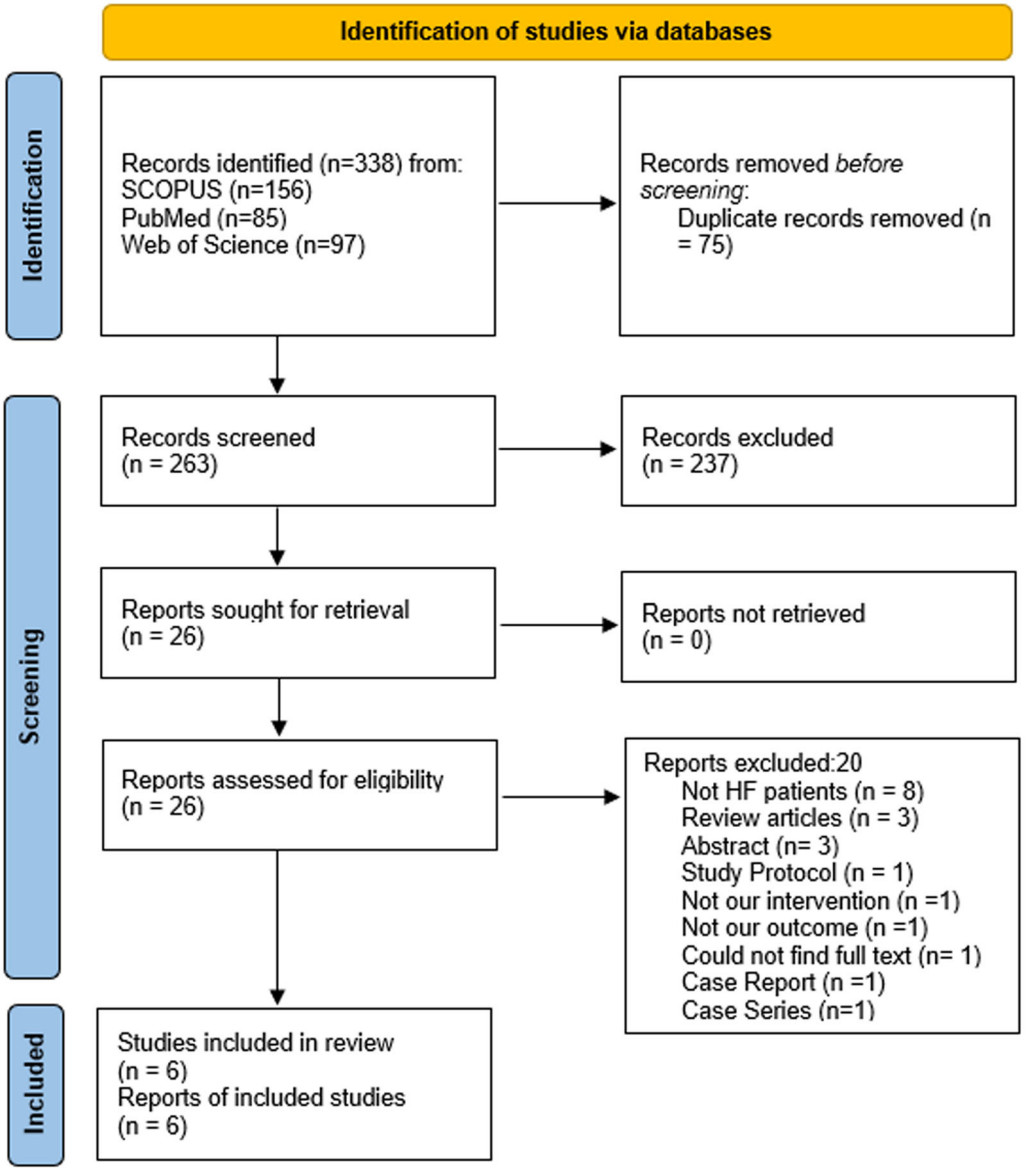
The Preferred Reporting Items for Systematic Reviews and Meta‐Analyses 2020 flow chart depicting the screening process for included studies.

We searched Scopus, Web of Science, and PubMed in January 2023. The search recovered 338 records, 75 of which were removed as duplicates. The remaining 263 studies qualified for the title and abstract screening, whereas only 26 met our inclusion criteria. Consequently, they were included in the full‐text screening. Finally, our systematic review and meta‐analysis featured six studies. The PRISMA diagram illustrating the steps of study selection is presented in Figure 1.

### 3.2 Baseline characteristics of included studies

Our systematic review included a total of six recently published studies (2018–2023), five of which were retrospective,^15^, ^16^, ^17^, ^18^, ^19^ and the last included a study by Pruszkowska et al.,^20^ l was a nonrandomized clinical trial. Patients with AF comprised a total of 1699 with a mean time for follow‐up of 19.76 months. An overview of included studies can be found in Table 1.

**Table 1:** Summary of the included studies.

Study ID	Study design	Country	Number of patients (*n*)	Follow‐up period (months)	Main inclusion criteria
Chen et al.^15^	Retrospective cohort study	China	471	23.1	Patients with: (i) drug‐resistant paroxysmal AF (PAF) or persistent AF (PersAF) who underwent CBA‐based pulmonary vein isolation (PVI) for the first time; (ii) HF diagnosed by cardiologists at each institution plus a documented history of HF.
Heeger et al.^16^	Retrospective case control study	Germany	100	12	Patients with PAF or PersAF and HF with LVEF ≤ 40% scheduled for CB2‐based PVI.
Pott et al.^17^	Retrospective cohort study	Germany	414	19.2	Patients who successfully underwent first‐time PVI for the treatment of symptomatic paroxysmal or persistent AF.
Prabhu et al.^18^	Retrospective single‐center cohort	Australia	76	28.1	Patients were included if they: (1) had documented preprocedural left ventricular systolic dysfunction (LVEF) ≤ 45% as determined by either echocardiography or cardiac MR within 6 months of the index procedure; (2) underwent index catheter ablation for AF incorporating de novo PV isolation with cryoballoon (CRYO).
Pruszkowska et al.^20^	Nonrandomised controlled trial	Poland	89	20.8	HF patients with LVEF ≤ 40% and previously implanted dual chamber ICD who were referred for ablation of paroxysmal or persistent AF between September 2012 and May 2015.
Yanagisawa et al.^19^	Retrospective large‐scale multicenter cohort	Japan	549	25.7	Patients with HF undergoing cryoballoon ablation for AF in 2014–2019 at the participating hospitals.

Abbreviations: AF, atrial fibrillation; CBA, cryoballoon ablation; HF, heart failure; ICD, implantable cardioverter‐defibrillator; lVEF, left ventricular ejection fraction; MR, magnetic resonance; PV, pulmonary vein.

The mean age of the patients with HFrEF was 65.42 (SD: 11.25), with 274 males and a mean body mass index of 24.69 (SD: 4.63). Among comorbidities, HTN was more common in HFrEF patients included. Baseline characteristics of all included studies are presented in Table S1.

### 3.3 Procedural data

Procedural time from a puncture to session end in all included studies ranged from 90 to 126 min, with persistent AF percentage ranging from 24% to 71% (Table S1). Only one study by Yanagisawa et al.^19^ included data on repeat ablation.

### 3.4 Assessment of the potential for bias

**Figure 2:**
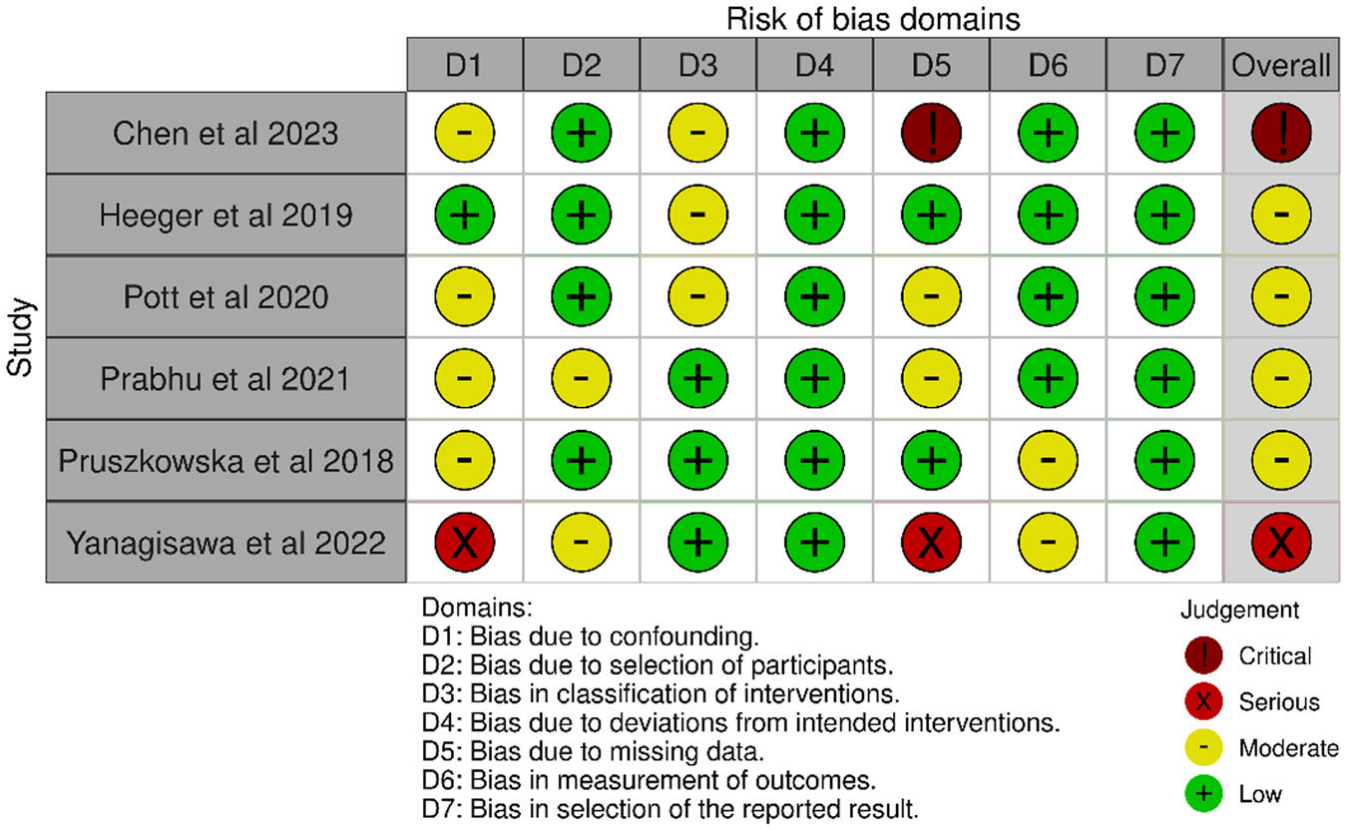
Quality assessment of included studies using ROBINS‐1 tool.

The quality assessment utilized the ROBINS‐1 tool to assess selection bias, confounding bias, intervention classification bias, incomplete outcome data, outcome measurement, and reporting bias. Four studies showed moderate overall bias,^16^, ^17^, ^18^, ^20^ while one was serious,^19^ and the last one was critical.^15^ Figure 2 shows the detailed quality assessment done on all the included studies.

### 3.5 Change in LVEF and NYHA functional class after cryoablation

**Figure 3:**
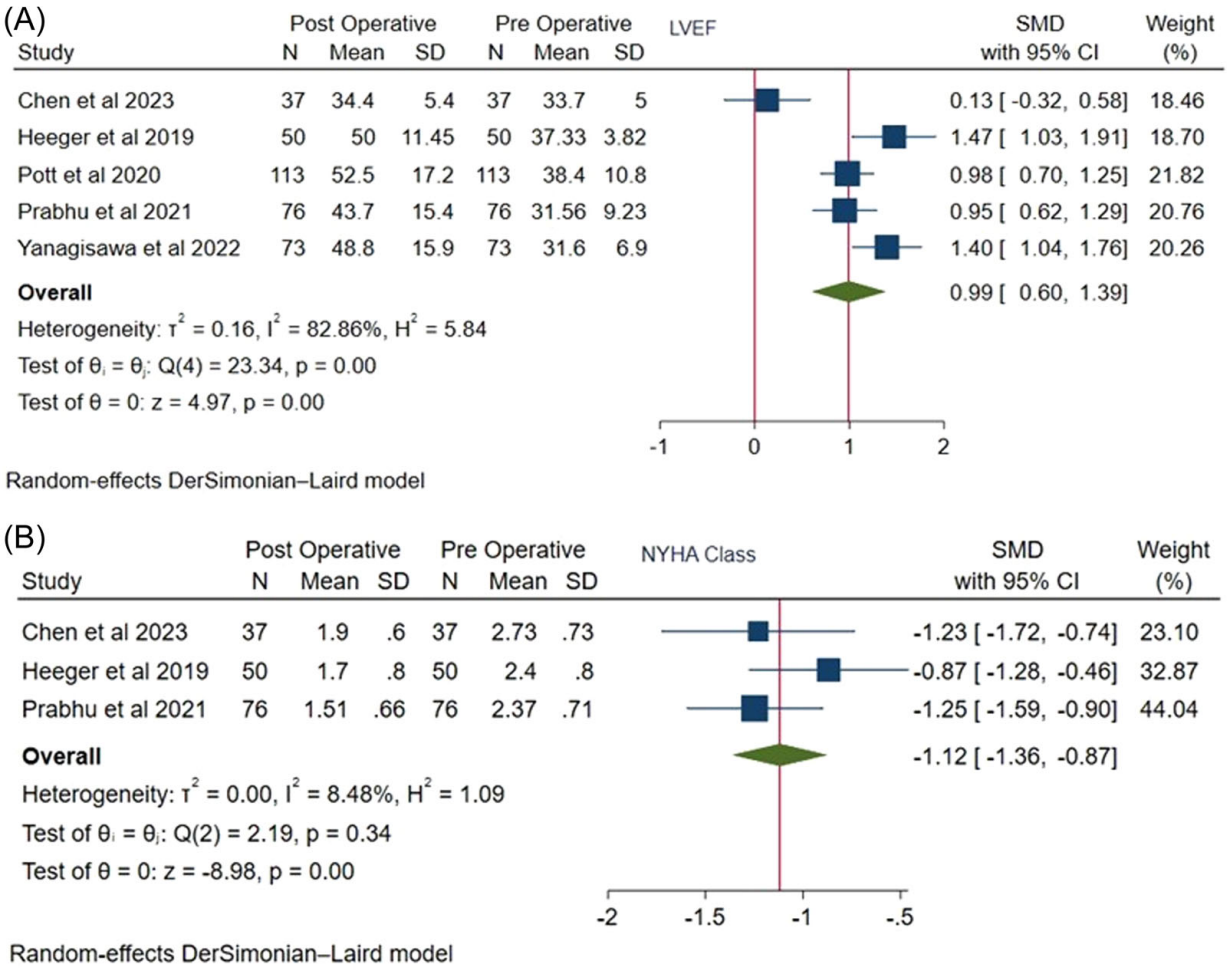
(A) Forest plot showing the change in left ventricular ejection fraction (LVEF) after successful ablation. (B) Forest plot showing the change in New York Heart Association functional class after successful. CI, confidence interval; SMD, standard mean difference.

The SMD of post and preoperative LVEF in HFrEF was 0.99 ([95% CI: 0.60, 1.39], *I* ^2^ = 82.86%, *p* = .00) (Figure 3A). Further, the SMD for improvement in NYHA class was −1.12 ([95% CI: −1.36, −0.87], *I* ^2^: 8.48%, *p* = .00) (Figure 3B).

### 3.6 Freedom from AF recurrence

**Figure 4:**
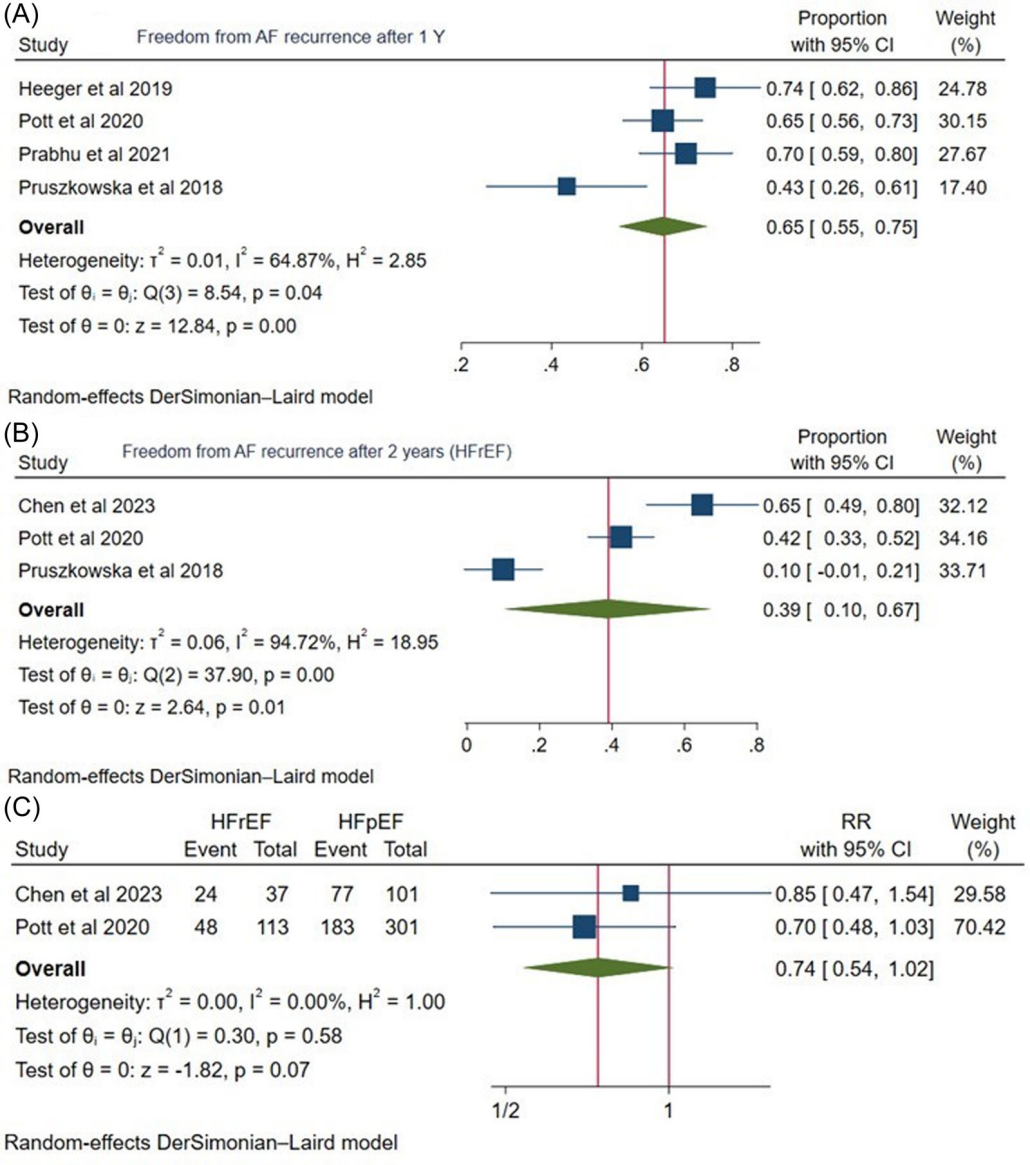
(A) Forest plot for freedom from atrial fibrillation after 1 year in HFrEF. (B) Forest plot for freedom from atrial fibrillation recurrence after 2 years in HFrEF. (C) Freedom from atrial fibrillation recurrence after 2 years of cryoablation in comparison between the two groups of HFrEF and HFpEF. AF, atrial fibrillation; CI, confidence interval; HFrEF, heart failure with reduced ejection fraction; HFpEF, heart failure with preserved ejection fraction.

The success rate was assessed using freedom from AF recurrence in HFrEF patients. Freedom from AF after 1 year of the procedure was 65% ([95% CI: 0.55, 0.75], *I* ^2^: 64.87%), and for AF after 2 years was 39% ([95% CI: 0.10, 0.67]), *I* ^2^: 94.72%) (Figure 4A,B).

After 2 years, a head‐to‐head comparison of HFrEF and HFpEF regarding freedom from AF revealed comparable results (risk ratio [RR]: 0.74 [95% CI: 0.54, 1.02], *I* ^2^: 0.00%, *p* = .07) (Figure 4C)

### 3.7 Secondary outcomes

#### 3.7.1 All‐cause death

Proportional analysis was conducted for all‐cause death resulting in 3% ([95% CI: −0.01, 0.07], *I* ^2^: 71.65%) (File S2).

#### 3.7.2 Complications

The complications of cryoablation observed in the HFrEF groups in five of the included studies were phrenic nerve palsy (the most commonly encountered complication in almost all included studies with seven cases out of 306 HFrEF patients), periprocedural transient ischemic attack (three reported case only from a total of 306 patients), and cardiac tamponade (only two cases out of 193 patients) (Table S2).

#### 3.7.3 Regression analysis

Regression analysis used continuous variables like comorbidities (HTN, DM, CAD), sex, age, follow‐up period, and persistent AF. There was no significant association between the outcomes and the mentioned variables.

#### 3.7.4 Publication bias and sensitivity analysis

The funnel plot for the primary outcome was qualitatively symmetrical with no small‐study effects assessed using the regression‐based Eggers test. Using the trim and fill command in STATA 17.0 for funnel plot did not address further probable studies for publication bias. After excluding one study at a time, sensitivity analysis utilized the leave‐one‐out method, which showed no statistical difference with the overall outcome except for freedom from AF after 2 years. Figures for funnel plot and sensitivity analysis are provided in File S3.

## 4 DISCUSSION

We conducted a systematic review of a total of six studies^15^, ^16^, ^17^, ^18^, ^19^, ^20^ with 365 HFrEF patients. Our findings significantly improved LVEF and NYHA classification in HFrEF patients (*p* = .00). Moreover, our results indicated a noteworthy effect of cryoablation in mitigating AF recurrence, alongside a success rate, denoted by freedom from AF at 1 and 2 years postprocedure.

It is well known that AF has negative consequences, particularly an increased risk of stroke. Recent studies have broadened this understanding, showing that AF also heightens mortality risk, HF, myocardial infarction, and hospital admissions. A prospective cohort study involving more than 15 000 patients with AF found that HF was the primary cause of death in 11% of cases in 1 year.^21^ In a large‐scale meta‐analysis with over 10 million participants, AF doubled the risk of HF and increased mortality risk fivefold.^22^

The debate over the extent to which a substantially decreased LVEF affects the improvement of HF through AF ablation continues. The AMICA trial revealed no enhancement in outcomes for patients with congestive HF post‐AF ablation.^23^ However, the study only included participants with chronic or long‐standing persistent AF. The CASTLE‐AF study showed the benefits of AF ablation in individuals with HF regarding the combined primary endpoint of HF hospitalization and death. Nevertheless, favorable outcomes in subgroup analysis were only observed in AF patients with LVEF equal to or less than 25%.^6^

Studies from our analysis supported these findings, demonstrating improvement in LVEF and NYHA classification. For instance, in the study by Pruszkowska et al.,^20^ patients with LVEF ≤ 40% showed enhancements in NYHA class and LVEF, elevating from 30% at baseline to 37% after 6 months (*p* = .007). Heeger et al.,^16^ utilizing CBA on HF patients, reported an increase in LVEF from a median of 37%–55% after a year of follow‐up (*p* < .0001). Noteworthy is that even among the recurrence group, Yanagisawa et al.^19^ documented a significant uplift in LVEF in the HFrEF group (from 30.9% to 43.6%, *p* = .006). Another investigation recorded an enhancement of LVEF from a mean of 31.56%–43.7% with SMD = 0.95, which closely aligns with our findings.^18^

Our findings are consistent with the meta‐analysis by AlTurki et al.^24^ that noted an improved LVEF following catheter ablation (weighted mean difference, 7.48; 95% CI: 3.71–11.26; *p* < .0001). This meta‐analysis, however, combined both CBA and RFA in the analysis, with a larger fraction of included studies employing RFA. Additionally, Mao et al.^25^ noted a significant reduction in arrhythmia recurrence postcatheter ablation compared to medical treatment in patients with reduced LVEF (RR: 0.42 [0.30, 0.60], *p* < .00001), yet cryoballoon was not specifically addressed in this meta‐analysis. Our analysis demonstrated an overall postoperative improvement in LVEF with an SMD of 0.99. We observed a consensus that CBA increased the EF to varying degrees when we compared it to the individual analysis performed in each included study. Chen et al.^15^ documented the lowest effect size (SMD = 0.13, [95% CI: −0.32, 0.58]), though favoring CBA in HFrEF, with the minor difference likely attributable to the limited sample size utilized.

Pruszkowska et al.^20^ observed a significant reduction in the AF burden postprocedure in patients with HFrEF (18.5% vs. 52.9%; *p* = .001). Following a single CBA treatment, Prabhu et al.^18^ reported that 70% of patients maintained freedom from atrial arrhythmia recurrence over 12 months. A comparable early recurrence rate of atrial arrhythmia was identified among patient groups with no HF, HFpEF, HFmrEF, and HFrEF (16.2% vs. 15.4% vs. 14.9% vs. 12.2%, *p* = .798).^15^ Chen et al.^15^ used univariate and multivariate logistic regression models to evaluate the recovery from HF. Regardless of the subtypes, the absence of AF within a year of CBA was a reliable indicator of HF recovery. These outcomes support our findings, indicating a satisfactory success rate and potentially improved prognosis in CBA patients, with negligible variation based on LVEF level in HF. At long‐term follow‐up, Prabhu et al.^18^ reported no statistically significant difference in procedure success rates after cryoablation between patients with paroxysmal AF and persistent AF (52% vs. 41%, *p* = .36). This was further confirmed in a multicentric study, which found no significant difference in AF recurrence after cryoablation between paroxysmal and persistent AF group (*p* = .964).^19^

AlTurki et al.^24^ showed that mortality decreased significantly following catheter ablation for AF in patients with HFrEF. However, all studies included in the AlTurki review were randomized controlled trials, different from our approach, and used mainly single‐arm studies. Mao et al.^25^ supported using CA to reduce mortality. Nevertheless, they specified that it was limited primarily to the subgroup of HF patients with a moderately decreased LVEF.

This novel study highlights the promising potential of CBA as a treatment option for this specific patient population. It is sensible to plan for future large‐scale studies to evaluate the outcomes of cryoablation in AF patients with HFrEF, using double‐arm studies with RF ablation as the comparator. Cryoablation holds great promise, particularly for patients with multiple comorbidities; however, further investigation is required to identify potential risks or complications.

## 5 STRENGTHS AND LIMITATIONS

One of the strengths of our meta‐analysis is that we included recent studies (2018–2023). Regarding including only studies testing the efficacy of CBA in HFrEF patients, our systematic review and meta‐analysis appear to be a first, with no previous meta‐analyses using the same criteria. As a result, our study stands out as a unique contribution to the field, as no other meta‐analyses specifically address the outcomes of AF recurrence or improvement of NYHA functional class and LVEF in AF patients with HFrEF following CBA. The sources of heterogeneity were thoroughly investigated using random effect models and a leave‐one‐out test for sensitivity analysis, which revealed no statistically significant differences in results. A funnel plot analysis revealed no evidence of publication bias. To the best of our knowledge, this is the first review to cover mostly single‐arm studies of patients with AF who have HFrEF and are undergoing cryoablation.

The primary limitation of this study is the inherent limitation of a single‐arm meta‐analysis in the absence of a control group for comparison. Furthermore, only a few studies were included due to a lack of research in the specified target group. Pott et al.^17^ reported freedom from atrial arrhythmia only after the procedure off AAD; however, it was included due to limited available data. Finally, the quality assessment of the included studies revealed a moderate to critical risk of bias, lowering our confidence in the results.

## 6 CONCLUSION

Cryoablation proved safety and efficacy in HFrEF, significantly improving NYHA class and LVEF and decreasing AF recurrence. Thus, this patient group could benefit from this treatment modality. However, randomized controlled trials are needed to validate these findings in HFrEF and investigate the long‐term effects of CBA in HFmrEF and HFpEF.

## AUTHOR CONTRIBUTIONS

Amira M. Taha got the study idea, performed the data collection, led and supervised the team, edited and peer reviewed the final manuscript. Nada I. Hendi, Ahmed B. Elwekel, Ahmed Atia, and Mohamed Elbanna all participated in the screening, data extraction, and quality assessment of the studies included. Nada I. Hendi participated in draft writing. Nouran A. Taha participated in writing and editing of the whole manuscript. Abhigan B. Shrestha performed the data analysis and interpretation. All authors have read and agreed to the final version of the manuscript.

## CONFLICT OF INTEREST STATEMENT

The authors declare no conflict of interest.

## Supporting information


**Supplementary 1: Search strategy**.

**Supplementary 2:** Forest plot showing the proportional analysis of mortality rate after ablation.

**Supplementary 3: Funnel plot and sensitivity analysis figures**.

**Supplementary Table 1: Baseline characteristics; and AF and procedural data of the included studies**.

**Supplementary Table 2: Complications of the procedure among included studies**.

## Data Availability

The data that support the findings of this study are available from the corresponding author upon reasonable request. All data generated or analyzed during this study are included in this published article or the data repositories listed in the references.
