# Supplementary material for: Cryoballoon ablation for atrial fibrillation in patients with heart failure and reduced left ventricular ejection fraction: A systematic review and meta‐analysis
Source: Clin Cardiol. 2023 Oct 25;47(1):e24177. doi: 10.1002/clc.24177 (PMC10766134; doi:10.1002/clc.24177)
Supplement: Supplementary file 1 — Supplementary 1: Search strategy. [file CLC-47-e24177-s001.docx]

**WOS**

1. ALL=(“Atrial Fibrillation” OR “Atrial Fibrillations” OR “AF”)
2. ALL=(“Cryosurgery” OR “Cryotherapy” OR “Cryoballoon” OR “Cryothermal”)
3. ALL=(“Heart failure” OR “Cardiac failure” OR “Heart decompensation” OR “Myocardial failure”)
4. #1 AND #2 AND #3

Results: 97

Date of search: 29 January 2023

No limitations applied

**PUBMED:**

1. ("Atrial Fibrillation"[All Fields] OR "Atrial Fibrillations"[All Fields] OR "AF"[All Fields])
2. ("Cryosurgery"[All Fields] OR "Cryotherapy"[All Fields] OR "Cryoballoon"[All Fields] OR "Cryothermal"[All Fields])
3. ("Heart failure"[All Fields] OR "Cardiac failure"[All Fields] OR "Heart decompensation"[All Fields] OR "Myocardial failure"[All Fields])

Results: 85

Date of search: 29 January 2023

No limitations applied

**SCOPUS:**

1. TITLE-ABS-KEY ( "cryosurgery" OR "cryotherapy" OR "cryoballoon" OR "cryothermal" )
2. TITLE-ABS-KEY ( "heart failure" OR "cardiac failure" OR "heart decompensation" OR "myocardial failure" )
3. TITLE-ABS-KEY ( "atrial fibrillation" OR "atrial fibrillations" OR "af" )
4. #1 AND #2 AND #3

Results: 156

Date of search: 29 January 2023

No limitations applied
