# Supplementary figures and images for: Cryoballoon ablation for atrial fibrillation in patients with heart failure and reduced left ventricular ejection fraction: A systematic review and meta‐analysis
Source: Clin Cardiol. 2023 Oct 25;47(1):e24177. doi: 10.1002/clc.24177 (PMC10766134; doi:10.1002/clc.24177)

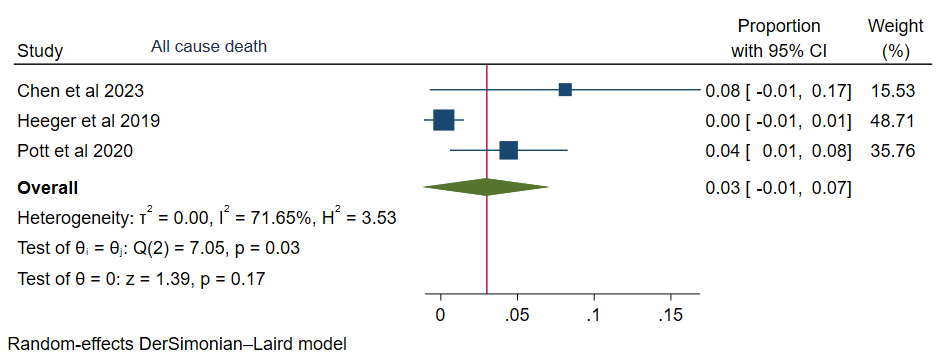

Supplement: Supplementary file 2 — Supplementary 2: Forest plot showing the proportional analysis of mortality rate after ablation. [file CLC-47-e24177-s005.docx]
