# Supplementary material for: Cryoballoon ablation for atrial fibrillation in patients with heart failure and reduced left ventricular ejection fraction: A systematic review and meta‐analysis
Source: Clin Cardiol. 2023 Oct 25;47(1):e24177. doi: 10.1002/clc.24177 (PMC10766134; doi:10.1002/clc.24177)
Supplement: Supplementary file 3 — Supplementary 3: Funnel plot and sensitivity analysis figures. [file CLC-47-e24177-s004.docx]

**Cryoballoon ablation for atrial fibrillation in patients with heart failure and reduced left ventricular ejection fraction: a systematic review and meta-analysis**

**Supplementary File-3**

**
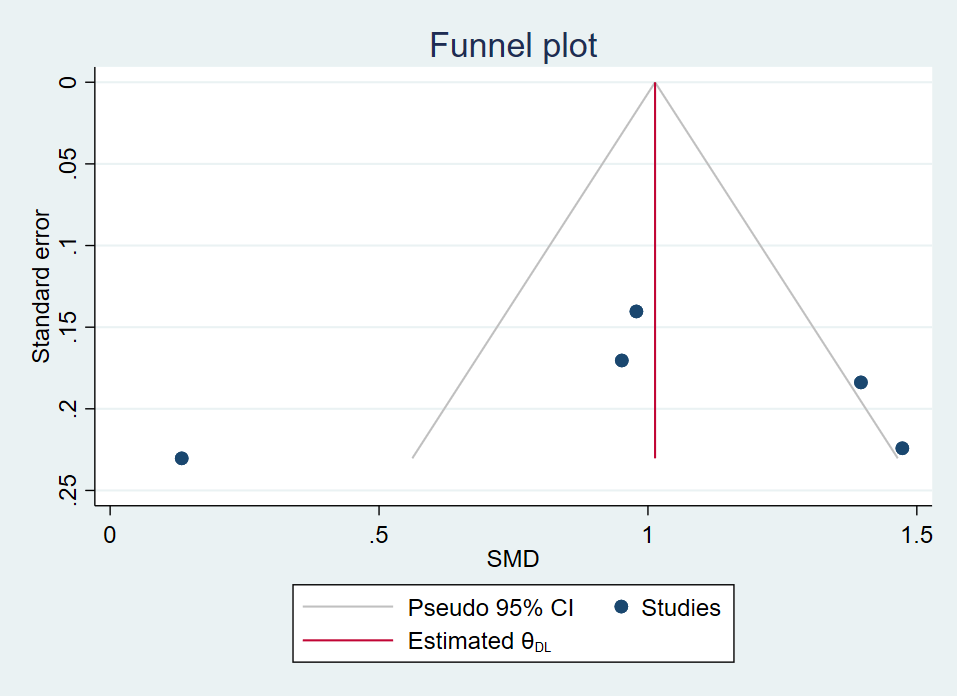
**

**Figure S-1: Funnel plot of the change in Left Ventricular Ejection Fraction (LVEF) after ablation.**

**
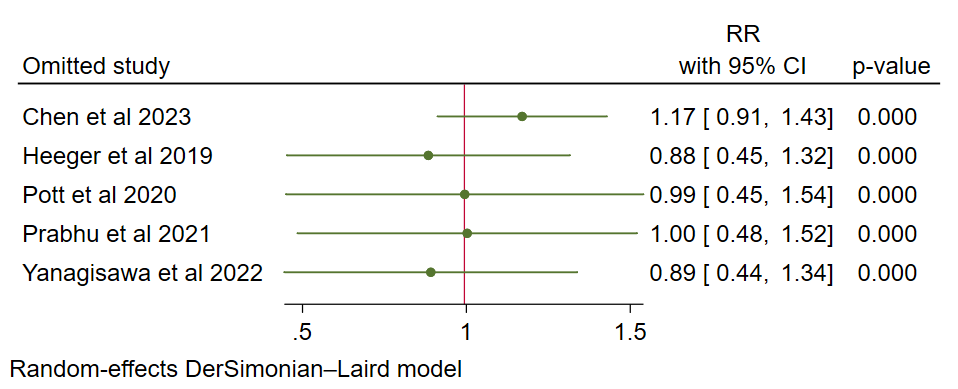
**

**Figure S-2: Sensitivity analysis of the change in Left Ventricular Ejection Fraction (LVEF) after ablation.**

**
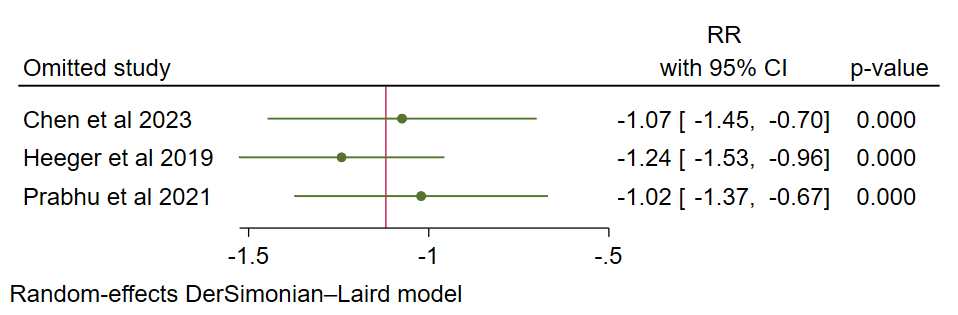
**

**Figure S-3: Sensitivity analysis of the change in New York Heart Association (NYHA) functional class after ablation**

**
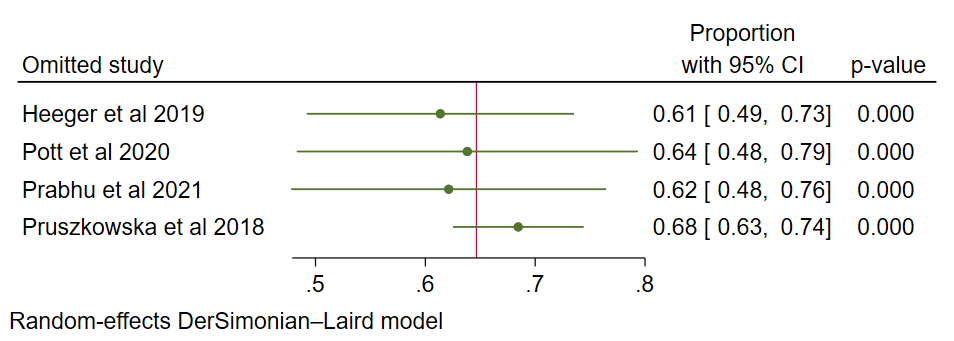
**

**Figure S-4: Sensitivity analysis of the freedom from atrial fibrillation after one year in HFrEF.**

**
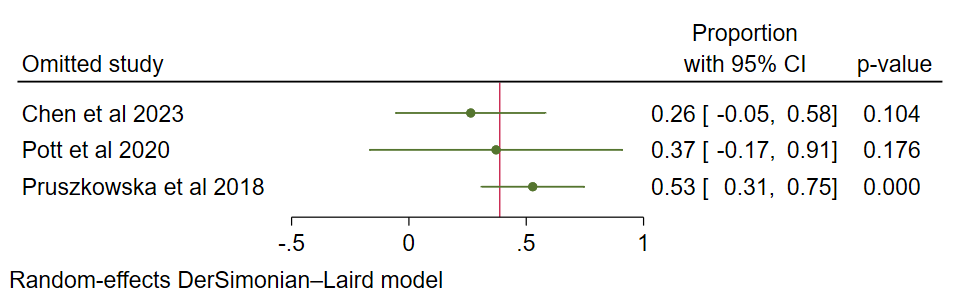
**

**Figure S-5: Sensitivity analysis of the freedom from atrial fibrillation recurrence after two years in HFrEF.**

**
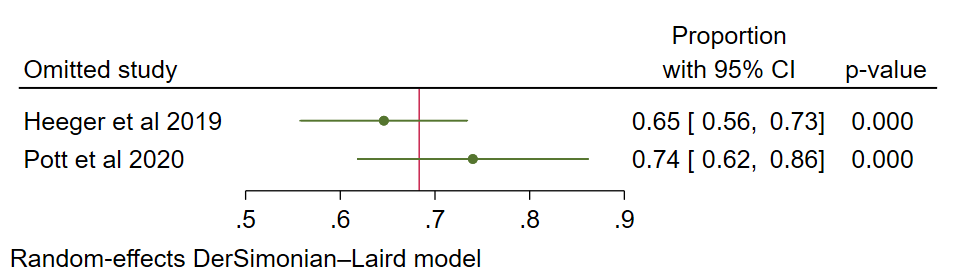
**

**Figure S-6: Sensitivity analysis of the proportional analysis of mortality rate after ablation.**
