# Supplementary material for: Cryoballoon ablation for atrial fibrillation in patients with heart failure and reduced left ventricular ejection fraction: A systematic review and meta‐analysis
Source: Clin Cardiol. 2023 Oct 25;47(1):e24177. doi: 10.1002/clc.24177 (PMC10766134; doi:10.1002/clc.24177)
Supplement: Supplementary file 4 — Supplementary Table 1: Baseline characteristics; and AF and procedural data of the included studies. [file CLC-47-e24177-s002.docx]

| **Study ID** | **Group** | **Age (M/SD)** | **Male**  **(n, %)** | **BMI**  **kg/m2**  **(M/SD)** | **Underlying diseases (n, %)** | | | | **Medication (n, %)** | | | |
| --- | --- | --- | --- | --- | --- | --- | --- | --- | --- | --- | --- | --- |
|  |  |  |  |  | **HTN** | **DM** | **CAD** | **Prior TIA/stroke** | **Beta blocker** | **Anti arrhythmic** | **Oral anticoagulants** | **ARB/ ACEI** |
| **Chen et al 2023(15)** | HFrEF | 68.33 ± 10.02 | 23 (62.2) | 22.67 ± 4.6 | 24 (64.9) | 13 (35.1) | 15 (40.5) | 3 (8.1) | 24 (64.9) | 7(18.2) | 31 (83.8) | 26 (70.2) |
|  | HFpEF | 66.33 ± 16.55 | 60 (59.4) | 23.1 ± 4.4 | 25 (31.6) | 28 (27.7) | 30 (29.7) | 3 (3) | 45 (44.6) | 22 (21.8) | 76 (75.2) | 55 (54.5)) |
|  | HFmrEF | 69.67 ± 7.55 | 48 (61.5) | 23.67 ± 5.5 | 46 (59) | 13 (35.1) | 28 (35.9) | 6 (7.7) | 44 (56.4) | 13 (16.7) | 64 (82.1) | 48 (61.5) |
|  | No HF | 64.33 ± 17.15 | 152 (59.6) | 22.38 ± 4.25 | 109 (42.7) | 50 (19.6) | 81 (31.8) | 1 (0.4) | 98 (38.4) | 37(14.5) | 139 (54.9) | 84 (32.9) |
| **Heeger et al 2019(16)** | HFrEF | 66 ± 11 | 34 (68) | 27.9 ± 3.6 | 38 (76) | 11 (50) | 15 (30) | 1 6 (12) | NR | NR | NR | NR |
|  | No HF | 65.6 ± 9 | 35 (70) | NR | 39 (78) | 11 (50) | 13 (26) | 5 (10) | NR | NR | NR | NR |
| **Pott et al 2020(17)** | HFrEF | 67.6 ± 10.2 | 73 (64.6) | 27.8 ± 5 | 99 (87.6) | 35 (31.0) | 58 (51.3) | NR | NR | NR | NR | NR |
|  | HFpEF | 65.9 ± 10.6 | 150(49,8) | 29.2 ± 5.7 | 235 (78.1) | 42 (14.0) | 100 (33.2) | NR | NR | NR | NR | NR |
| **Prabhu et al 2021(18)** | HFrEF | 63 ± 11 | 61(80) | NR | 17(21) | 8(10.5) | 28 (37) | NR | 59(78) | 41(54) | 24(32) | 69(91) |
| **Pruszkowska et al 2018(20)** | HFrEF | 63 ± 13 | 25 (83) | NR | 16 (53) | 16 (53) | 15 (50) | NR | NR | NR | NR | NR |
|  | No HF | 60 ± 11 | 28 (47) | NR | 35 (59) | 4 (7) | 8 (14) | NR | NR | NR | NR | NR |
| **Yanagisawa et al 2022(19)** | HFrEF | 64.6 ± 12.3 | 58 (80) | 23.6 ± 4.3 | 35 (48) | 22 (30) | 16 (22) | 11 (15) | 58 (80) | 11 (15), | 60 (82) | 38 (52) |
|  | HFpEF | 70.3 ± 9.4 | 202 (51) | 23.9 ± 3.8 | 262 (67) | 72 (18) | 26 (6.6) | 43 (11) | 247 (63) | 91 (23) | 349 (89) | 182 (46) |
|  | HFmrEF | 68.6 ± 9.4 | 55 (67) | 23.3 ± 3.7 | 46 (56) | 25 (30) | 19 (23) | 7 (8.5) | 67 (82) | 8 (9.8) | 74 (90) | 46 (56) |

**Abbreviations: BMI**=body mass index, **HTN**=hypertension, **DM**= diabetes mellitus, **CAD**= carotid artery disease, **TIA**= transient ischemic attack, **ACEI**= angiotensin converting enzyme inhibitors, **ARB**=angiotensin receptor blocker, **HFpEF**= heart failure with preserved ejection fraction, **HFrEF**= heart failure with reduced ejection fraction, **HFmrEF**= heart failure with mildly reduced ejection fraction, **HF**=heart failure, **NR**= not recorded, **m**= months.

| **Study ID** | **Group** | **Procedure time (mean minutes ± SD)** | **Duration of AF prior to intervention (mean years ± SD)** | **AF Type (n, %)** | | | **No. of isolated Pulmonary Veins (n, %)** | **repeated ablation**  **(n, %)** |
| --- | --- | --- | --- | --- | --- | --- | --- | --- |
|  |  |  |  | **Paroxysmal** | **Persistent** | **L.S. P** |  |  |
| **Chen et al. 2023** | HFrEF | 122.3(22.3) | 1.11(0.45) | NR | 9(24.3) | NR | 142(96) | NR |
|  | HFpEF | 116.6(15.3) | 1.025(0.25) | NR | 27(26.7) | NR | 395(97.77) | NR |
|  | HFmrEF | 119(13.5) | 0.94(0.44) | NR | 17(21.8) | NR | 304(97.4) | NR |
|  | No HF | 111.7(14.8) | 0.89(0.38) | NR | 47(18.4) | NR | 999( 97.9) | NR |
| **Heeger et al. 2019** | HFrEF | 131(39) | 1.42(2.1) | NR | 14(28) | NR | 197(99) | NR |
|  | No HF | 126(33) | 1.6(2.42) | NR | 13(26) | NR | 197(99) | NR |
| **Pott et al. 2020** | HFrEF | 98.3(31.6) | NR | NR | 62 (54.9) | NR | NR | NR |
|  | HFpEF | 103.7(37) | NR | NR | 75 (24.9) | NR | NR | NR |
| **Prabhu et al. 2021** | HFrEF | 90(42) | NR | 59(29) | 146(71) | 54(26) | 300( 99) | 10(13) |
| **Pruszkowska et al. 2018** | HFrEF | 110(35) | NR | 16(53) | 14(47) | NR | NR | NR |
|  | No HF | 115(45) | NR | 51(86) | 8(14) | NR | NR | NR |
| **Yanagisawa et al. 2022** | HFrEF | 126(48) | 1.17(1.34) | 40(55) | 28(38) | 5(6.8) | NR | 102(19) |
|  | HFpEF | 136(37) | 0.9(1.36) | 397(75) | 84(21) | 13(3.3) | NR | 102(19) |
|  | HFmrEF | 132(46) | 0.7(0.8) | 54(66) | 22(27) | 6(7.3) | NR | 102(19) |

**Abbreviations:L.S.P:** Long‐standing persistent, **NR**= not recorded, **HFpEF**= heart failure with preserved ejection fraction, **HFrEF**= heart failure with reduced ejection fraction, **HFmrEF**= heart failure with mildly reduced ejection fraction, **HF**=heart failure, **AF**=atrial fibrillation.
