# Supplementary material for: Cryoballoon ablation for atrial fibrillation in patients with heart failure and reduced left ventricular ejection fraction: A systematic review and meta‐analysis
Source: Clin Cardiol. 2023 Oct 25;47(1):e24177. doi: 10.1002/clc.24177 (PMC10766134; doi:10.1002/clc.24177)
Supplement: Supplementary file 5 — Supplementary Table 2: Complications of the procedure among included studies. [file CLC-47-e24177-s003.docx]

| **Study ID** | **Group** | **Complications** (**n, %)** | | | |
| --- | --- | --- | --- | --- | --- |
|  |  | **Phrenic Nerve Palsy** | **Periprocedural TIA/Stroke** | **Pericardial Effusion** | **Cardiac Tamponade** |
| **Chen et al. 2023** | HFrEF | 0 | 3 (8.1) | NR | 0 |
| **Heeger et al. 2019** | HFrEF | 3(6) | 0 | 0 | 1(2) |
| **Pott et al. 2020** | HFrEF | 3(2.7) | 0 | 0 | NR |
| **Prabhu et al. 2021** | HFrEF | 0 | 0 | NR | 0 |
| **Pruszkowska et al. 2018** | HFrEF | 1(3.3) | 0 | 0 | 1(3.3) |
| **Yanagisawa et al. 2022** | HFrEF | NR | NR | NR | NR |

**Supplementary table 2: Procedure complications**

**Abbreviations: NR**= not recorded, **HFpEF**= heart failure with preserved ejection fraction, **HFrEF**= heart failure with reduced ejection fraction, **HFmrEF**= heart failure with mildly reduced ejection fraction, **HF**=heart failure, **TIA**= transient ischemic attack.
